# Tetra­aqua­bis­[1-(3-carb­oxy­phen­yl)-4,4′-bipyridin-1-ium-κ*N* ^1′^]zinc bis­(4,5-carb­oxy­benzene-1,2-dicarboxyl­ate) 2.5-hydrate

**DOI:** 10.1107/S1600536811045156

**Published:** 2011-11-05

**Authors:** Jie Zhang, Yi Tan, Zhiyong Fu

**Affiliations:** a Key Lab for Fuel Cell Technology of Guangdong Province, School of Chemistry and Chemical Engineering, South China University of Technology, Guangzhou, People’s Republic of China

## Abstract

In the complex cation of the title compound, [Zn(C_17_H_13_N_2_O_2_)_2_(H_2_O)_4_](C_10_H_4_O_8_)_2_·2.5H_2_O, the Zn^II^ atom, lying on an inversion center, is coordinated by two N atoms from two *N*-(3-carb­oxy­phen­yl)-4,4′-bipyridin-1-ium ligands and four water mol­ecules in a distorted octa­hedral geometry. The pyromellitate anion is double deprotonated. O—H⋯O and C—H⋯O hydrogen bonds connect the cations, anions and uncoordinated water mol­ecules into a three-dimensional supra­molecular network. One of the two lattice water molecules shows an occupancy of 0.25. An intra­molecular O—H⋯O hydrogen bond is present in the anion.

## Related literature

For background to the structures and applications of viologen compounds, see: Ebbesen *et al.* (1984 ▶); Jin *et al.* (2010 ▶); Sun *et al.* (2005 ▶); Xu *et al.* (2007 ▶).
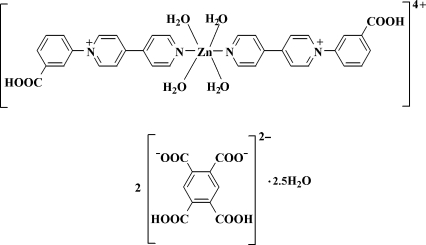


## Experimental

### 

#### Crystal data

[Zn(C_17_H_13_N_2_O_2_)_2_(H_2_O)_4_](C_10_H_4_O_8_)_2_·2.5H_2_O
*M* *_r_* = 1241.33
Monoclinic, 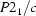

*a* = 7.5476 (2) Å
*b* = 19.5528 (4) Å
*c* = 19.2752 (4) Å
β = 110.431 (2)°
*V* = 2665.63 (10) Å^3^
*Z* = 2
Mo *K*α radiation
μ = 0.56 mm^−1^
*T* = 298 K
0.43 × 0.41 × 0.37 mm

#### Data collection

Bruker APEX CCD diffractometer
Absorption correction: multi-scan (*SADABS*; Sheldrick, 1996 ▶) *T*_min_ = 0.781, *T*_max_ = 0.825
25451 measured reflections
4867 independent reflections
4250 reflections with *I* > 2σ(*I*)
*R*_int_ = 0.028

#### Refinement

*R*[*F* ^2^ > 2σ(*F* ^2^)] = 0.031
*wR*(*F* ^2^) = 0.078
*S* = 1.06
4867 reflections
429 parameters
4 restraints
H atoms treated by a mixture of independent and constrained refinement
Δρ_max_ = 0.29 e Å^−3^
Δρ_min_ = −0.36 e Å^−3^

### 

Data collection: *SMART* (Bruker, 2007 ▶); cell refinement: *SAINT* (Bruker, 2007 ▶); data reduction: *SAINT*; program(s) used to solve structure: *SHELXS97* (Sheldrick, 2008 ▶); program(s) used to refine structure: *SHELXL97* (Sheldrick, 2008 ▶); molecular graphics: *DIAMOND* (Brandenburg, 1999 ▶); software used to prepare material for publication: *SHELXTL* (Sheldrick, 2008 ▶).

## Supplementary Material

Crystal structure: contains datablock(s) global, I. DOI: 10.1107/S1600536811045156/hy2468sup1.cif

Structure factors: contains datablock(s) I. DOI: 10.1107/S1600536811045156/hy2468Isup2.hkl

Additional supplementary materials:  crystallographic information; 3D view; checkCIF report

## Figures and Tables

**Table 1:** Hydrogen-bond geometry (Å, °)

*D*—H⋯*A*	*D*—H	H⋯*A*	*D*⋯*A*	*D*—H⋯*A*
O1—H7⋯O12^i^	0.87 (2)	1.80 (3)	2.656 (3)	164 (3)
O3—H1⋯O11^ii^	0.85 (2)	1.88 (3)	2.732 (2)	175 (3)
O3—H2⋯O11^iii^	0.81 (3)	2.03 (2)	2.820 (4)	163 (2)
O4—H3⋯O13^iii^	0.84 (2)	1.84 (3)	2.677 (6)	175 (3)
O4—H4⋯O12^iv^	0.90 (3)	1.79 (4)	2.694 (2)	177 (4)
O6—H9⋯O7	0.87 (3)	1.56 (3)	2.418 (2)	174 (3)
O9—H8⋯O8^iii^	0.85 (3)	1.82 (3)	2.646 (3)	163 (4)
O13—H5⋯O10	0.92 (4)	1.90 (2)	2.813 (3)	174 (3)
O13—H6⋯O7^iii^	0.87 (4)	1.86 (4)	2.723 (2)	169 (3)
O14—H11⋯O13	0.82 (2)	2.18 (2)	2.846 (2)	138 (2)
C7—H7*A*⋯O1^v^	0.93	2.30	3.155 (2)	153
C8—H8*A*⋯O12^vi^	0.93	2.52	3.302 (2)	142
C9—H9*A*⋯O2^vii^	0.93	2.30	3.225 (2)	172
C10—H10*A*⋯O5^viii^	0.93	2.15	3.060 (3)	167

Symmetry codes: (i) 
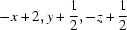
; (ii) 
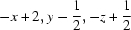
; (iii) 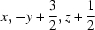; (iv) 
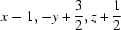
; (v) 
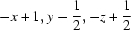
; (vi) 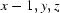; (vii) 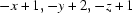; (viii) 
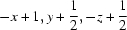
.

**Fig. 1.**
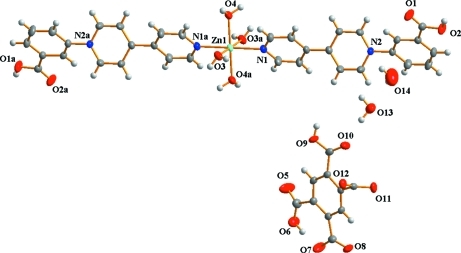
The molecular structure of the title compound. Displacement ellipsoids are drawn at the 50% probability level. [Symmetry codes: (a) 1-x, 1-y, 1-z.]

**Fig. 2.**
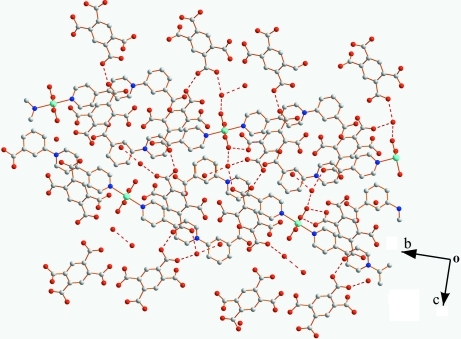
The three-dimensional supramolecular structure of the title compound. Dashed lines denote hydrogen bonds.

## supplementary crystallographic information

### Comment

Viologens are quaternary salts derived from 4,4'-bipyridine (Ebbesen *et al.*, 1984). Intensively interests have been focused on these compounds for their electron-transfer properties and enormous potential applications in electrochromic displays and optically switchable devices (Jin *et al.*, 2010; Sun *et al.*, 2005; Xu *et al.*, 2007). Most of them are dimethyl-, diethyl-, dibetaine- and dibenzyl viologens. Here we report the synthesis and structure of a new viologen compound with *N*-3-carboxyphenyl substitution and double-deprotonated pyromellitate as compensation anions.

The asymmetric unit of the title compound contains one Zn^II^ atom lying on an inversion center, one *N*-(3-carboxyphenyl)-4,4'-bipyridinium ligand, one double-deprotonated pyromellitate anion, two coordinated water molecules and 1.25 uncooedinated water molecules (Fig. 1). The Zn^II^ atom is six-coordinated by two N atoms of the viologen ligands and four O atoms of water molecules. The Zn—N bond length is 2.1689 (14) Å and the Zn—O bond lengths are 2.0798 (13) and 2.1335 (14) Å. The bond angles vary from 88.98 (6) to 93.42 (5)°, indicating a distorted octahedral geometry. O—H···O and C—H···O hydrogen bonds (Table 1) connect the cations, anions and uncoordinated water molecules into a three-dimensional supramolecular network (Fig. 2).

### Experimental

A mixture of *N*-(3-carboxyphenyl)-4,4'-bipyridinium chloride (0.2 mmol), ZnO(1 mmol), phosphoric acid (2 mmol), pyromellitic acid (0.2 mmol) and water (5 ml) was sealed in a 23 ml Teflon-lined bomb at 120°C for 72 h. Yellow block-shaped crystals were obtained.

### Refinement

H atoms of the hydroxyl groups and water molecules were located in a difference Fourier map and refined with *U*_iso_(H) = 1.5*U*_eq_(O). H atoms attached to O6 and O14 were refined with a distance restraint of O—H = 0.82 (2) Å and with *U*_iso_(H) = 1.5*U*_eq_(O). Other H atoms were positioned geometrically and refined using a riding model, with C—H = 0.93 Å and with *U*_iso_(H) = 1.2*U*_eq_(C).

### Crystal data

**Table:**

[Zn(C_17_H_13_N_2_O_2_)_2_(H_2_O)_4_](C_10_H_4_O_8_)_2_·2.5H_2_O	*F*(000) = 1282
*M**_r_* = 1241.33	*D*_x_ = 1.547 Mg m^−^^3^
Monoclinic, *P*2_1_/*c*	Mo *K*α radiation, λ = 0.71073 Å
Hall symbol: -P 2ybc	Cell parameters from 4867 reflections
*a* = 7.5476 (2) Å	θ = 3.1–25.4°
*b* = 19.5528 (4) Å	µ = 0.56 mm^−^^1^
*c* = 19.2752 (4) Å	*T* = 298 K
β = 110.431 (2)°	Block, yellow
*V* = 2665.63 (10) Å^3^	0.43 × 0.41 × 0.37 mm
*Z* = 2	

### Data collection

**Table:**

Bruker APEX CCD diffractometer	4867 independent reflections
Radiation source: fine-focus sealed tube	4250 reflections with *I* > 2σ(*I*)
graphite	*R*_int_ = 0.028
φ and ω scans	θ_max_ = 25.4°, θ_min_ = 3.1°
Absorption correction: multi-scan (*SADABS*; Sheldrick, 1996)	*h* = −9→8
*T*_min_ = 0.781, *T*_max_ = 0.825	*k* = −23→23
25451 measured reflections	*l* = −23→23

### Refinement

**Table:**

Refinement on *F*^2^	Primary atom site location: structure-invariant direct methods
Least-squares matrix: full	Secondary atom site location: difference Fourier map
*R*[*F*^2^ > 2σ(*F*^2^)] = 0.031	Hydrogen site location: inferred from neighbouring sites
*wR*(*F*^2^) = 0.078	H atoms treated by a mixture of independent and constrained refinement
*S* = 1.06	*w* = 1/[σ^2^(*F*_o_^2^) + (0.0331*P*)^2^ + 1.443*P*] where *P* = (*F*_o_^2^ + 2*F*_c_^2^)/3
4867 reflections	(Δ/σ)_max_ = 0.001
429 parameters	Δρ_max_ = 0.29 e Å^−^^3^
4 restraints	Δρ_min_ = −0.36 e Å^−^^3^

### Fractional atomic coordinates and isotropic or equivalent isotropic displacement parameters (Å^2^)

**Table:**

	*x*	*y*	*z*	*U*_iso_*/*U*_eq_	Occ. (<1)
Zn1	0.5000	0.5000	0.5000	0.02391 (9)	
O1	0.5186 (2)	1.24085 (7)	0.33078 (8)	0.0434 (4)	
H7	0.583 (4)	1.2716 (14)	0.3626 (15)	0.065*	
O2	0.6136 (2)	1.16786 (7)	0.42474 (8)	0.0513 (4)	
O3	0.80086 (19)	0.50774 (7)	0.53939 (8)	0.0327 (3)	
H1	0.844 (3)	0.4741 (13)	0.5221 (13)	0.049*	
H2	0.855 (3)	0.5404 (13)	0.5307 (13)	0.049*	
O4	0.4919 (2)	0.53685 (7)	0.60006 (8)	0.0344 (3)	
H3	0.591 (4)	0.5432 (13)	0.6368 (14)	0.052*	
H4	0.423 (3)	0.5747 (13)	0.5993 (13)	0.052*	
O5	0.7710 (4)	0.55277 (9)	0.05365 (11)	0.0879 (8)	
O6	0.7924 (2)	0.53366 (8)	−0.05325 (9)	0.0507 (4)	
H9	0.819 (4)	0.5542 (14)	−0.0882 (13)	0.076*	
O7	0.8657 (3)	0.59789 (8)	−0.14700 (9)	0.0702 (6)	
O8	0.9161 (2)	0.70368 (8)	−0.17320 (7)	0.0464 (4)	
O9	0.9545 (2)	0.74468 (7)	0.20592 (7)	0.0419 (4)	
H8	0.939 (4)	0.7691 (13)	0.2399 (15)	0.063*	
O10	0.9735 (2)	0.84566 (7)	0.15526 (8)	0.0444 (4)	
O11	1.04737 (18)	0.89852 (6)	0.00847 (7)	0.0349 (3)	
O12	1.29187 (17)	0.84798 (6)	0.09494 (7)	0.0313 (3)	
O13	0.8157 (3)	0.95061 (10)	0.21503 (10)	0.0555 (4)	
H5	0.872 (5)	0.9152 (19)	0.199 (2)	0.105 (13)*	
H6	0.816 (5)	0.9357 (17)	0.257 (2)	0.097 (12)*	
O14	0.8680 (18)	1.0750 (5)	0.2965 (6)	0.109 (3)	0.25
H10	0.91 (3)	1.062 (10)	0.340 (4)	0.163*	0.25
H11	0.798 (10)	1.044 (3)	0.275 (10)	0.163*	0.25
N1	0.4598 (2)	0.60196 (7)	0.45273 (8)	0.0267 (3)	
N2	0.3534 (2)	0.93790 (7)	0.31154 (8)	0.0253 (3)	
C1	0.3529 (3)	0.61428 (9)	0.38216 (11)	0.0359 (4)	
H1A	0.2941	0.5775	0.3525	0.043*	
C2	0.3255 (3)	0.67884 (9)	0.35108 (11)	0.0360 (4)	
H2A	0.2528	0.6848	0.3014	0.043*	
C3	0.5401 (3)	0.65598 (9)	0.49434 (10)	0.0299 (4)	
H3A	0.6167	0.6485	0.5433	0.036*	
C4	0.5151 (3)	0.72233 (9)	0.46828 (10)	0.0301 (4)	
H4A	0.5702	0.7584	0.4999	0.036*	
C5	0.4079 (2)	0.73488 (8)	0.39504 (10)	0.0253 (4)	
C6	0.3860 (2)	0.80580 (9)	0.36554 (10)	0.0256 (4)	
C7	0.3945 (3)	0.81976 (9)	0.29649 (10)	0.0340 (4)	
H7A	0.4100	0.7841	0.2672	0.041*	
C8	0.3803 (3)	0.88552 (9)	0.27086 (10)	0.0343 (4)	
H8A	0.3893	0.8942	0.2248	0.041*	
C9	0.3598 (3)	0.86071 (9)	0.40711 (10)	0.0284 (4)	
H9A	0.3547	0.8532	0.4540	0.034*	
C10	0.3416 (3)	0.92569 (9)	0.37883 (10)	0.0288 (4)	
H10A	0.3208	0.9619	0.4063	0.035*	
C12	0.3409 (3)	1.00795 (8)	0.28392 (10)	0.0263 (4)	
C13	0.2324 (3)	1.02160 (10)	0.21080 (10)	0.0355 (4)	
H13A	0.1684	0.9866	0.1793	0.043*	
C14	0.2213 (3)	1.08835 (10)	0.18574 (11)	0.0407 (5)	
H14A	0.1504	1.0983	0.1367	0.049*	
C15	0.3149 (3)	1.14040 (9)	0.23309 (10)	0.0330 (4)	
H15A	0.3052	1.1852	0.2160	0.040*	
C16	0.4233 (3)	1.12578 (9)	0.30622 (10)	0.0261 (4)	
C17	0.4371 (2)	1.05870 (9)	0.33179 (9)	0.0265 (4)	
H17A	0.5103	1.0483	0.3805	0.032*	
C18	0.5277 (3)	1.17943 (9)	0.36038 (10)	0.0290 (4)	
C19	0.8122 (3)	0.57302 (10)	0.00281 (12)	0.0367 (5)	
C20	0.8992 (3)	0.65999 (10)	−0.13012 (10)	0.0311 (4)	
C21	0.9692 (2)	0.78374 (9)	0.15265 (10)	0.0272 (4)	
C22	1.1268 (2)	0.84837 (8)	0.04705 (9)	0.0240 (4)	
C23	0.8857 (2)	0.64591 (9)	0.00489 (10)	0.0252 (4)	
C24	0.9082 (2)	0.67918 (9)	0.07170 (9)	0.0262 (4)	
H24A	0.8778	0.6557	0.1080	0.031*	
C25	0.9740 (2)	0.74564 (9)	0.08605 (9)	0.0231 (4)	
C26	1.0261 (2)	0.78040 (8)	0.03287 (9)	0.0223 (3)	
C27	0.9965 (2)	0.74906 (9)	−0.03482 (9)	0.0245 (4)	
H27A	1.0256	0.7733	−0.0710	0.029*	
C28	0.9254 (2)	0.68298 (9)	−0.05120 (9)	0.0237 (4)	

### Atomic displacement parameters (Å^2^)

**Table:**

	*U*^11^	*U*^22^	*U*^33^	*U*^12^	*U*^13^	*U*^23^
Zn1	0.03018 (16)	0.01496 (15)	0.02675 (16)	0.00253 (11)	0.01013 (12)	0.00130 (11)
O1	0.0654 (10)	0.0207 (7)	0.0339 (8)	−0.0122 (7)	0.0046 (7)	0.0038 (6)
O2	0.0864 (12)	0.0321 (8)	0.0254 (8)	−0.0156 (8)	0.0070 (8)	0.0014 (6)
O3	0.0322 (7)	0.0228 (7)	0.0456 (8)	−0.0012 (6)	0.0167 (6)	−0.0025 (6)
O4	0.0456 (8)	0.0273 (7)	0.0299 (7)	0.0110 (6)	0.0125 (6)	−0.0006 (6)
O5	0.178 (2)	0.0368 (10)	0.0895 (14)	−0.0447 (12)	0.0982 (16)	−0.0147 (9)
O6	0.0767 (11)	0.0299 (8)	0.0470 (9)	−0.0207 (8)	0.0234 (8)	−0.0105 (7)
O7	0.1403 (18)	0.0379 (9)	0.0362 (9)	−0.0227 (10)	0.0359 (10)	−0.0152 (7)
O8	0.0765 (11)	0.0419 (9)	0.0260 (7)	−0.0017 (8)	0.0243 (7)	0.0016 (6)
O9	0.0714 (10)	0.0359 (8)	0.0269 (7)	−0.0035 (7)	0.0281 (7)	−0.0002 (6)
O10	0.0751 (11)	0.0284 (8)	0.0404 (8)	−0.0017 (7)	0.0335 (8)	−0.0051 (6)
O11	0.0391 (7)	0.0211 (7)	0.0442 (8)	0.0020 (6)	0.0141 (6)	0.0095 (6)
O12	0.0334 (7)	0.0211 (6)	0.0359 (7)	−0.0047 (5)	0.0076 (6)	0.0014 (5)
O13	0.0707 (11)	0.0586 (11)	0.0343 (9)	0.0133 (9)	0.0147 (8)	0.0069 (8)
O14	0.142 (9)	0.066 (6)	0.087 (7)	0.003 (6)	0.001 (7)	−0.007 (5)
N1	0.0324 (8)	0.0173 (7)	0.0303 (8)	0.0015 (6)	0.0108 (7)	0.0009 (6)
N2	0.0356 (8)	0.0162 (7)	0.0235 (7)	0.0000 (6)	0.0094 (6)	0.0008 (6)
C1	0.0510 (12)	0.0183 (9)	0.0312 (10)	−0.0001 (8)	0.0053 (9)	−0.0028 (8)
C2	0.0529 (12)	0.0205 (9)	0.0272 (10)	0.0028 (8)	0.0047 (9)	0.0012 (8)
C3	0.0334 (10)	0.0235 (9)	0.0282 (10)	−0.0008 (8)	0.0051 (8)	0.0033 (7)
C4	0.0363 (10)	0.0184 (9)	0.0313 (10)	−0.0048 (7)	0.0065 (8)	−0.0022 (7)
C5	0.0310 (9)	0.0172 (8)	0.0292 (9)	0.0023 (7)	0.0127 (8)	0.0018 (7)
C6	0.0294 (9)	0.0187 (9)	0.0270 (9)	−0.0001 (7)	0.0078 (7)	−0.0003 (7)
C7	0.0573 (12)	0.0190 (9)	0.0288 (10)	0.0031 (8)	0.0189 (9)	−0.0033 (7)
C8	0.0582 (12)	0.0233 (9)	0.0255 (10)	0.0008 (9)	0.0198 (9)	−0.0006 (8)
C9	0.0421 (10)	0.0215 (9)	0.0242 (9)	0.0005 (8)	0.0148 (8)	0.0013 (7)
C10	0.0428 (11)	0.0201 (9)	0.0268 (9)	0.0015 (8)	0.0162 (8)	−0.0028 (7)
C12	0.0360 (10)	0.0174 (9)	0.0263 (9)	0.0006 (7)	0.0119 (8)	0.0035 (7)
C13	0.0464 (11)	0.0254 (10)	0.0271 (10)	−0.0056 (8)	0.0032 (9)	−0.0015 (8)
C14	0.0528 (13)	0.0314 (11)	0.0254 (10)	−0.0017 (9)	−0.0021 (9)	0.0072 (8)
C15	0.0443 (11)	0.0201 (9)	0.0312 (10)	−0.0003 (8)	0.0091 (9)	0.0070 (8)
C16	0.0340 (9)	0.0198 (9)	0.0260 (9)	0.0000 (7)	0.0123 (8)	0.0021 (7)
C17	0.0342 (10)	0.0228 (9)	0.0217 (9)	0.0005 (7)	0.0089 (7)	0.0030 (7)
C18	0.0393 (10)	0.0227 (9)	0.0278 (10)	−0.0021 (8)	0.0154 (8)	0.0013 (7)
C19	0.0467 (12)	0.0244 (10)	0.0439 (12)	−0.0072 (9)	0.0221 (10)	−0.0011 (9)
C20	0.0374 (10)	0.0316 (10)	0.0240 (9)	0.0008 (8)	0.0104 (8)	−0.0018 (8)
C21	0.0280 (9)	0.0308 (10)	0.0239 (9)	−0.0018 (7)	0.0104 (7)	0.0005 (7)
C22	0.0314 (9)	0.0191 (9)	0.0260 (9)	−0.0012 (7)	0.0157 (8)	0.0000 (7)
C23	0.0278 (9)	0.0215 (9)	0.0267 (9)	−0.0029 (7)	0.0102 (7)	0.0004 (7)
C24	0.0311 (9)	0.0266 (9)	0.0240 (9)	−0.0022 (7)	0.0134 (7)	0.0048 (7)
C25	0.0245 (8)	0.0241 (9)	0.0203 (8)	−0.0009 (7)	0.0073 (7)	0.0010 (7)
C26	0.0243 (8)	0.0196 (8)	0.0232 (8)	0.0004 (7)	0.0087 (7)	0.0018 (7)
C27	0.0301 (9)	0.0227 (9)	0.0233 (9)	−0.0012 (7)	0.0124 (7)	0.0045 (7)
C28	0.0258 (9)	0.0234 (9)	0.0216 (9)	0.0006 (7)	0.0077 (7)	0.0006 (7)

### Geometric parameters (Å, °)

**Table:**

Zn1—O4	2.0798 (13)	C4—C5	1.382 (3)
Zn1—O3	2.1335 (14)	C4—H4A	0.9300
Zn1—N1	2.1689 (14)	C5—C6	1.486 (2)
O1—C18	1.321 (2)	C6—C7	1.382 (3)
O1—H7	0.88 (3)	C6—C9	1.394 (2)
O2—C18	1.204 (2)	C7—C8	1.368 (3)
O3—H1	0.85 (3)	C7—H7A	0.9300
O3—H2	0.81 (3)	C8—H8A	0.9300
O4—H3	0.84 (3)	C9—C10	1.371 (2)
O4—H4	0.90 (3)	C9—H9A	0.9300
O5—C19	1.195 (2)	C10—H10A	0.9300
O6—C19	1.292 (2)	C12—C17	1.377 (2)
O6—H9	0.867 (17)	C12—C13	1.387 (3)
O7—C20	1.260 (2)	C13—C14	1.384 (3)
O8—C20	1.229 (2)	C13—H13A	0.9300
O9—C21	1.315 (2)	C14—C15	1.385 (3)
O9—H8	0.85 (3)	C14—H14A	0.9300
O10—C21	1.212 (2)	C15—C16	1.390 (3)
O11—C22	1.250 (2)	C15—H15A	0.9300
O12—C22	1.266 (2)	C16—C17	1.392 (2)
O13—H5	0.92 (4)	C16—C18	1.496 (2)
O13—H6	0.87 (4)	C17—H17A	0.9300
O14—H10	0.82 (2)	C19—C23	1.525 (2)
O14—H11	0.82 (2)	C20—C28	1.531 (2)
N1—C3	1.337 (2)	C21—C25	1.496 (2)
N1—C1	1.339 (2)	C22—C26	1.508 (2)
N2—C8	1.348 (2)	C23—C24	1.399 (2)
N2—C10	1.352 (2)	C23—C28	1.418 (2)
N2—C12	1.460 (2)	C24—C25	1.384 (2)
C1—C2	1.382 (3)	C24—H24A	0.9300
C1—H1A	0.9300	C25—C26	1.396 (2)
C2—C5	1.392 (3)	C26—C27	1.387 (2)
C2—H2A	0.9300	C27—C28	1.392 (2)
C3—C4	1.380 (2)	C27—H27A	0.9300
C3—H3A	0.9300		
			
O4^i^—Zn1—O4	180.0	C10—C9—H9A	120.0
O4^i^—Zn1—O3^i^	91.02 (6)	C6—C9—H9A	120.0
O4—Zn1—O3^i^	88.98 (6)	N2—C10—C9	120.95 (16)
O4^i^—Zn1—O3	88.98 (6)	N2—C10—H10A	119.5
O4—Zn1—O3	91.02 (6)	C9—C10—H10A	119.5
O3^i^—Zn1—O3	180.0	C17—C12—C13	121.91 (16)
O4^i^—Zn1—N1	88.50 (5)	C17—C12—N2	118.68 (15)
O4—Zn1—N1	91.50 (5)	C13—C12—N2	119.41 (16)
O3^i^—Zn1—N1	86.58 (5)	C14—C13—C12	118.60 (17)
O3—Zn1—N1	93.42 (5)	C14—C13—H13A	120.7
O4^i^—Zn1—N1^i^	91.50 (5)	C12—C13—H13A	120.7
O4—Zn1—N1^i^	88.50 (5)	C13—C14—C15	120.55 (18)
O3^i^—Zn1—N1^i^	93.42 (5)	C13—C14—H14A	119.7
O3—Zn1—N1^i^	86.58 (5)	C15—C14—H14A	119.7
N1—Zn1—N1^i^	180.00 (8)	C14—C15—C16	120.09 (17)
C18—O1—H7	113.0 (17)	C14—C15—H15A	120.0
Zn1—O3—H1	107.9 (16)	C16—C15—H15A	120.0
Zn1—O3—H2	122.5 (17)	C15—C16—C17	119.83 (16)
H1—O3—H2	104 (2)	C15—C16—C18	122.93 (16)
Zn1—O4—H3	121.7 (17)	C17—C16—C18	117.23 (16)
Zn1—O4—H4	118.2 (15)	C12—C17—C16	119.02 (16)
H3—O4—H4	103 (2)	C12—C17—H17A	120.5
C19—O6—H9	112 (2)	C16—C17—H17A	120.5
C21—O9—H8	110.3 (18)	O2—C18—O1	122.97 (17)
H5—O13—H6	102 (3)	O2—C18—C16	123.40 (16)
H10—O14—H11	104 (10)	O1—C18—C16	113.63 (16)
C3—N1—C1	116.98 (15)	O5—C19—O6	120.08 (19)
C3—N1—Zn1	120.43 (12)	O5—C19—C23	119.00 (18)
C1—N1—Zn1	122.58 (12)	O6—C19—C23	120.91 (17)
C8—N2—C10	119.85 (15)	O8—C20—O7	123.68 (18)
C8—N2—C12	120.28 (14)	O8—C20—C28	117.35 (16)
C10—N2—C12	119.85 (14)	O7—C20—C28	118.95 (16)
N1—C1—C2	123.42 (17)	O10—C21—O9	123.79 (17)
N1—C1—H1A	118.3	O10—C21—C25	121.67 (16)
C2—C1—H1A	118.3	O9—C21—C25	114.51 (16)
C1—C2—C5	119.20 (17)	O11—C22—O12	125.74 (16)
C1—C2—H2A	120.4	O11—C22—C26	118.48 (15)
C5—C2—H2A	120.4	O12—C22—C26	115.63 (15)
N1—C3—C4	123.26 (17)	C24—C23—C28	118.29 (15)
N1—C3—H3A	118.4	C24—C23—C19	112.65 (15)
C4—C3—H3A	118.4	C28—C23—C19	129.02 (16)
C3—C4—C5	119.72 (17)	C25—C24—C23	122.71 (15)
C3—C4—H4A	120.1	C25—C24—H24A	118.6
C5—C4—H4A	120.1	C23—C24—H24A	118.6
C4—C5—C2	117.37 (16)	C24—C25—C26	118.93 (15)
C4—C5—C6	120.26 (16)	C24—C25—C21	121.64 (15)
C2—C5—C6	122.36 (16)	C26—C25—C21	119.03 (15)
C7—C6—C9	117.70 (16)	C27—C26—C25	118.78 (15)
C7—C6—C5	121.10 (15)	C27—C26—C22	117.73 (15)
C9—C6—C5	121.18 (16)	C25—C26—C22	123.32 (15)
C8—C7—C6	120.62 (17)	C26—C27—C28	123.08 (15)
C8—C7—H7A	119.7	C26—C27—H27A	118.5
C6—C7—H7A	119.7	C28—C27—H27A	118.5
N2—C8—C7	120.86 (17)	C27—C28—C23	117.96 (15)
N2—C8—H8A	119.6	C27—C28—C20	113.79 (15)
C7—C8—H8A	119.6	C23—C28—C20	128.25 (16)
C10—C9—C6	119.99 (16)		

Symmetry codes: (i) −*x*+1, −*y*+1, −*z*+1.

### Hydrogen-bond geometry (Å, °)

**Table:**

*D*—H···*A*	*D*—H	H···*A*	*D*···*A*	*D*—H···*A*
O1—H7···O12^ii^	0.87 (2)	1.80 (3)	2.656 (3)	164 (3)
O3—H1···O11^iii^	0.85 (2)	1.88 (3)	2.732 (2)	175 (3)
O3—H2···O11^iv^	0.81 (3)	2.03 (2)	2.820 (4)	163 (2)
O4—H3···O13^iv^	0.84 (2)	1.84 (3)	2.677 (6)	175 (3)
O4—H4···O12^v^	0.90 (3)	1.79 (4)	2.694 (2)	177 (4)
O6—H9···O7	0.87 (3)	1.56 (3)	2.418 (2)	174 (3)
O9—H8···O8^iv^	0.85 (3)	1.82 (3)	2.646 (3)	163 (4)
O13—H5···O10	0.92 (4)	1.90 (2)	2.813 (3)	174 (3)
O13—H6···O7^iv^	0.87 (4)	1.86 (4)	2.723 (2)	169 (3)
O14—H11···O13	0.82 (2)	2.18 (2)	2.846 (2)	138 (2)
C7—H7A···O1^vi^	0.93	2.30	3.155 (2)	153
C8—H8A···O12^vii^	0.93	2.52	3.302 (2)	142
C9—H9A···O2^viii^	0.93	2.30	3.225 (2)	172
C10—H10A···O5^ix^	0.93	2.15	3.060 (3)	167

Symmetry codes: (ii) −*x*+2, *y*+1/2, −*z*+1/2; (iii) −*x*+2, *y*−1/2, −*z*+1/2; (iv) *x*, −*y*+3/2, *z*+1/2; (v) *x*−1, −*y*+3/2, *z*+1/2;  (vi) −*x*+1, *y*−1/2, −*z*+1/2; (vii) *x*−1, *y*, *z*; (viii) −*x*+1, −*y*+2, −*z*+1; (ix) −*x*+1, *y*+1/2, −*z*+1/2.
